# A Knowledge-Enhanced Iterative Reasoning Framework for Accurate and Traceable Fault Diagnosis in Distributed Service Systems

**DOI:** 10.3390/s26175571

**Published:** 2026-09-02

**Authors:** Yuze Zhang, Jian Zhang, Junyuan Wang, Shan Zhang

**Affiliations:** 1 Swinburne College, Shandong University of Science and Technology, Jinan 250031, China; 2 College of Energy Engineering, Zhejiang University, Hangzhou 310027, China

**Keywords:** distributed service systems, large language model, knowledge graph, fault diagnosis

## Abstract

Fault diagnosis in distributed systems is challenged by complex service dependencies, cascading anomaly propagation, and similar symptom patterns. This paper proposes a knowledge-enhanced iterative reasoning framework that integrates large language models (LLMs) with a numerical domain knowledge graph (KG). The KG encodes fault–symptom relations, anomaly directions, and training-derived mean and standard-deviation intervals. Structured prompting first generates candidate faults; interval verification then rejects numerically inconsistent candidates. For retained candidates, counterfactual reasoning constructs hierarchical causal chains, KG traversal refines missing or inconsistent links, and a deterministic evidence score supports acceptance, exclusion, early stopping, and fallback across at most five iterations. Under the common 68-case evaluation protocol for eight known single-root-cause faults in the controlled Redis-based testbed, the complete framework achieved 100.00% Accuracy, Macro-F1, and Balanced Accuracy with GPT-4o and GPT-5.2, compared with 91.18% accuracy for KG-only reasoning and 85.29–89.71% for Random Forest, XGBoost, and Transformer baselines. GPT-3.5 reached 98.53%, whereas LLaMA-3.1-8B reached 80.88%, showing that the incremental KG–LLM gain is backbone-dependent. Five GPT-4o repetitions and three GPT-5.2 repetitions yielded 100.00% ± 0.00, and all three metrics remained at 100.00% across the evaluated Z-score thresholds, iteration limits, and interval tolerances. The framework therefore provides highly accurate, stable, and traceable diagnoses within the evaluated Redis-based distributed-service protocol, while providing explicit intermediate reasoning and solution retrieval.

## 1. Introduction

Cloud computing, the Internet of Things, and microservice architectures have made distributed services a central part of modern computing infrastructure. Their scale and inter-service dependencies also amplify reliability risks: a local resource, configuration, or communication anomaly can propagate through a service graph and produce system-wide symptoms [1,2,3]. Accurate fault localization and root-cause analysis are therefore essential for maintaining service continuity.

Existing diagnosis methods can be broadly grouped into rule/statistical, data-driven, knowledge-driven, and LLM-assisted approaches. Rule-based methods remain transparent but require extensive manual maintenance, while statistical approaches infer abnormal changes from monitored distributions. Isukapalli and Srirama surveyed fault-tolerant solutions for distributed analytics [4], and BARO applies multivariate Bayesian online change-point detection to robust microservice root-cause analysis [5]. These methods provide useful baselines, although high-dimensional and evolving service environments continue to challenge manually engineered features and fixed rules.

Data-driven methods learn fault patterns directly from monitoring data. Outage-Watch introduces extreme-event regularization for early outage prediction [6], while Transformer and temporal-convolution architectures model global and local dependencies in system logs [7]. Deep models can be highly accurate when training and test distributions are aligned, but their transfer to unseen topologies, workloads, or fault combinations remains a central concern [2,8].

Knowledge-driven methods explicitly model component dependencies, causal relations, and propagation paths. Liang et al. construct reusable meta-causal knowledge for cloud-native root-cause analysis [9], and KGroot combines a knowledge graph with graph learning to identify event associations and candidate root causes [10]. Such methods offer transparent constraints and traceable paths, but graph construction and maintenance can be costly, and purely graph-based rules may be insufficient when symptoms are ambiguous or partially observed.

The proposed framework is motivated by the iterative workflow used in expert diagnosis: observe anomalies, formulate a candidate, reason through plausible propagation paths, compare predicted effects with measured evidence, and revise the hypothesis when the evidence is insufficient. LLMs provide flexible semantic and causal reasoning, but they cannot independently guarantee access to current monitoring values or system-specific topology. The KG therefore supplies explicit domain constraints, while the LLM organizes candidate generation and causal-chain construction.

Recent fault-diagnosis research also provides strong data-driven alternatives. Physical fault testing with time–frequency networks and sparse-attention Transformers improves train suspension diagnosis [11]; deep adversarial capsule networks address multidomain generalization [12]; time–frequency fully connected graph neural networks learn multiscale dependencies from multisource measurements [13]; traceable algorithm-unrolling networks expose sparse diagnostic computations [14]; and robust weight-shared capsule networks improve machinery diagnosis under noise and limited data [15]. These methods motivate stronger comparisons in representation learning, generalization, and interpretability, but they do not provide the same combination of a numerical fault KG, counterfactual causal-chain refinement, and deterministic iterative verification developed here.

For LLM-assisted root-cause analysis, chaos-engineering evaluations show that unconstrained zero-shot diagnosis remains unreliable even when detailed context is supplied [16]. Domain knowledge and data fusion can improve multi-fault reasoning [17], and recent KG-enhanced LLM frameworks demonstrate the value of causal evidence chains in industrial and medical diagnosis [18,19]. The unresolved issue is how to couple flexible LLM reasoning with numerical, train-only KG constraints so that every accepted diagnosis has an explicit and reproducible evidence path.

To position the proposed framework relative to the most closely related approaches, we compare them along six methodological dimensions. Advanced data-driven diagnosis methods learn temporal or time-frequency representations but generally do not encode system-specific numerical fault intervals. Graph-based RCA methods explicitly represent components and causal relations, but most do not include an LLM-mediated counterfactual hypothesis-revision loop. Existing KG-enhanced LLM studies demonstrate the value of external knowledge and causal evidence, whereas the present framework further combines training-derived numerical constraints, iterative candidate exclusion, counterfactual causal-chain construction, KG-based chain correction, deterministic acceptance and early stopping, and an explicit fallback rule. Consequently, the originality of this work lies in the numerical KG-constrained iterative verification mechanism and the inspectable evidence path produced for each diagnosis. The current experimental scope is a controlled Redis-based distributed-service protocol with eight known fault classes.

Based on the above analysis, this paper proposes a fault diagnosis method for distributed systems that synergizes knowledge graphs and LLMs. The core idea of this method is to explicitly model component dependencies, fault propagation paths, and historical case knowledge using a knowledge graph, thereby providing structured domain knowledge support for LLMs [20]. At the same time, leveraging the semantic understanding and reasoning capabilities of LLMs, the method achieves flexible mapping from observed symptoms to candidate faults and simulates the closed-loop verification process of experts through a multi-stage iterative mechanism. Following this line of thinking, this paper focuses on addressing the following three key problems:

- Question 1: How are we to construct a high-quality knowledge graph covering system architecture, component dependencies, and fault propagation mechanisms to provide an explainable domain knowledge foundation for diagnosis?
- Question 2: How are we to design a collaborative mechanism between the LLMs and the knowledge graph, enabling effective interaction in stages such as fault hypothesis generation, causal chain reasoning, and confidence verification?
- Question 3: Can the framework provide accurate and traceable diagnoses across different LLM backbones under a controlled distributed-service fault protocol?

The main contributions are threefold. First, we construct a numerical fault-diagnosis KG that combines component–fault relations, symptom directions, class-specific mean intervals, and standard-deviation intervals calculated only from training windows. Second, we develop a four-stage iterative mechanism integrating candidate generation, interval verification, counterfactual causal-chain construction, KG refinement, and deterministic evidence-based acceptance or fallback. Third, we evaluate the framework under a common 68-case protocol against Random Forest, XGBoost, a temporal Transformer, KG-only reasoning, and four LLM backbones, including repeated executions, parameter sensitivity, and module-level ablations. The results demonstrate highly accurate and traceable diagnosis within the controlled Redis testbed and identify backbone reliability as an explicit condition for positive KG–LLM synergy.

## 2. Materials and Methods

**Figure 1:**
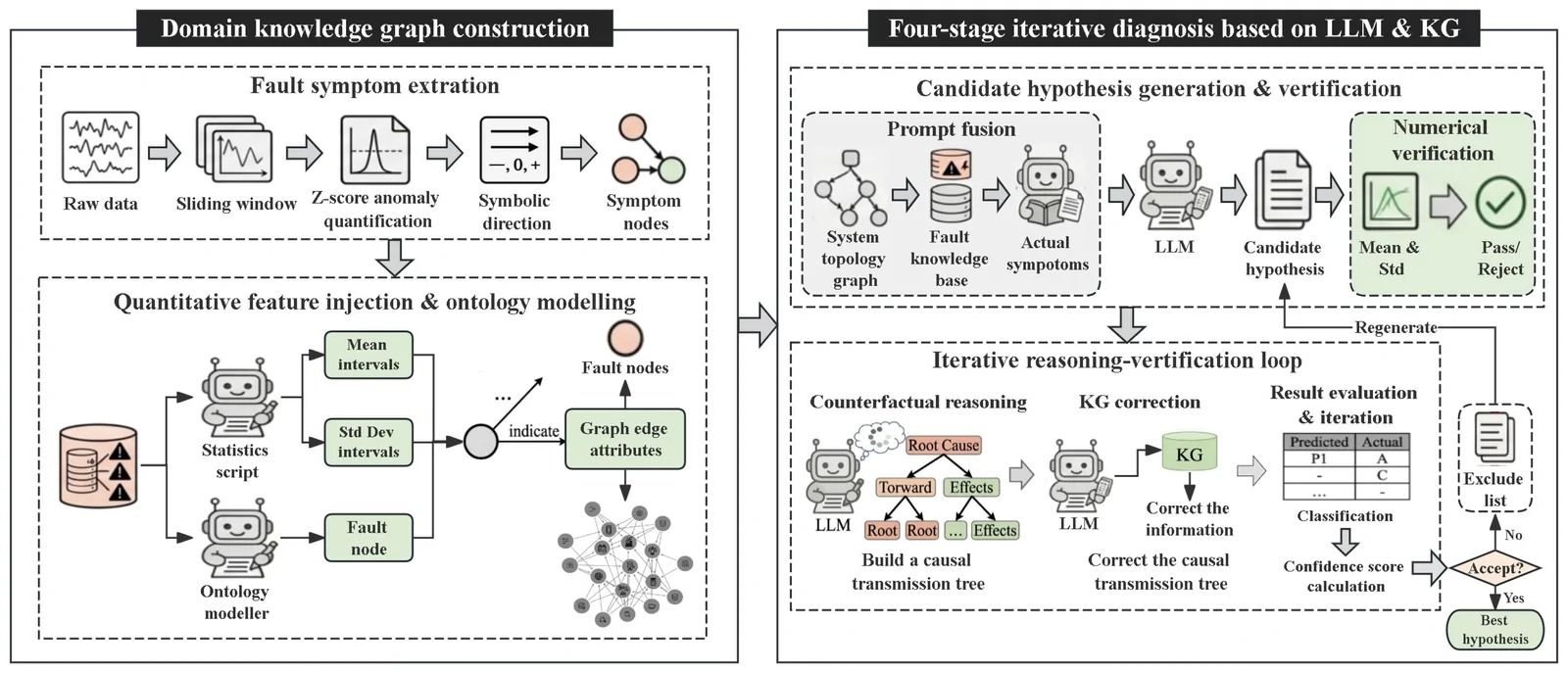
Flowchart of the LLM-based fault diagnosis system.

The fault diagnosis method proposed in this paper consists of two major components, which are domain knowledge graph construction and collaborative reasoning with LLMs. The overall system is illustrated in Figure 1. In the knowledge graph construction part, based on historical fault data, fault types and symptom metrics along with their directions and numerical intervals are organized into a structured ontology through window segmentation and symptom extraction, providing explicit and traceable knowledge support for subsequent reasoning. The LLM-KG collaborative reasoning component adopts an LLM-based iterative mechanism. In the first stage, candidate hypotheses are generated based on system mechanisms and fault knowledge. In the second stage, counterfactual causal reasoning is performed on the screened candidates to construct hierarchical propagation chains. In the third stage, the propagation chains are refined and supplemented using structured relations in the knowledge graph. In the fourth stage, predicted symptoms are compared with actual symptoms, confidence scores are computed, and the hypothesis is either accepted or iteratively eliminated. Through deep collaboration between the LLMs and the knowledge graph, this system achieves a complete cognitive loop from data perception to causal reasoning and from knowledge verification to iterative convergence, offering a high-accuracy and traceable diagnostic workflow for fault diagnosis in distributed systems.

### 2.1. Construction of the Domain KG

**Figure 2:**
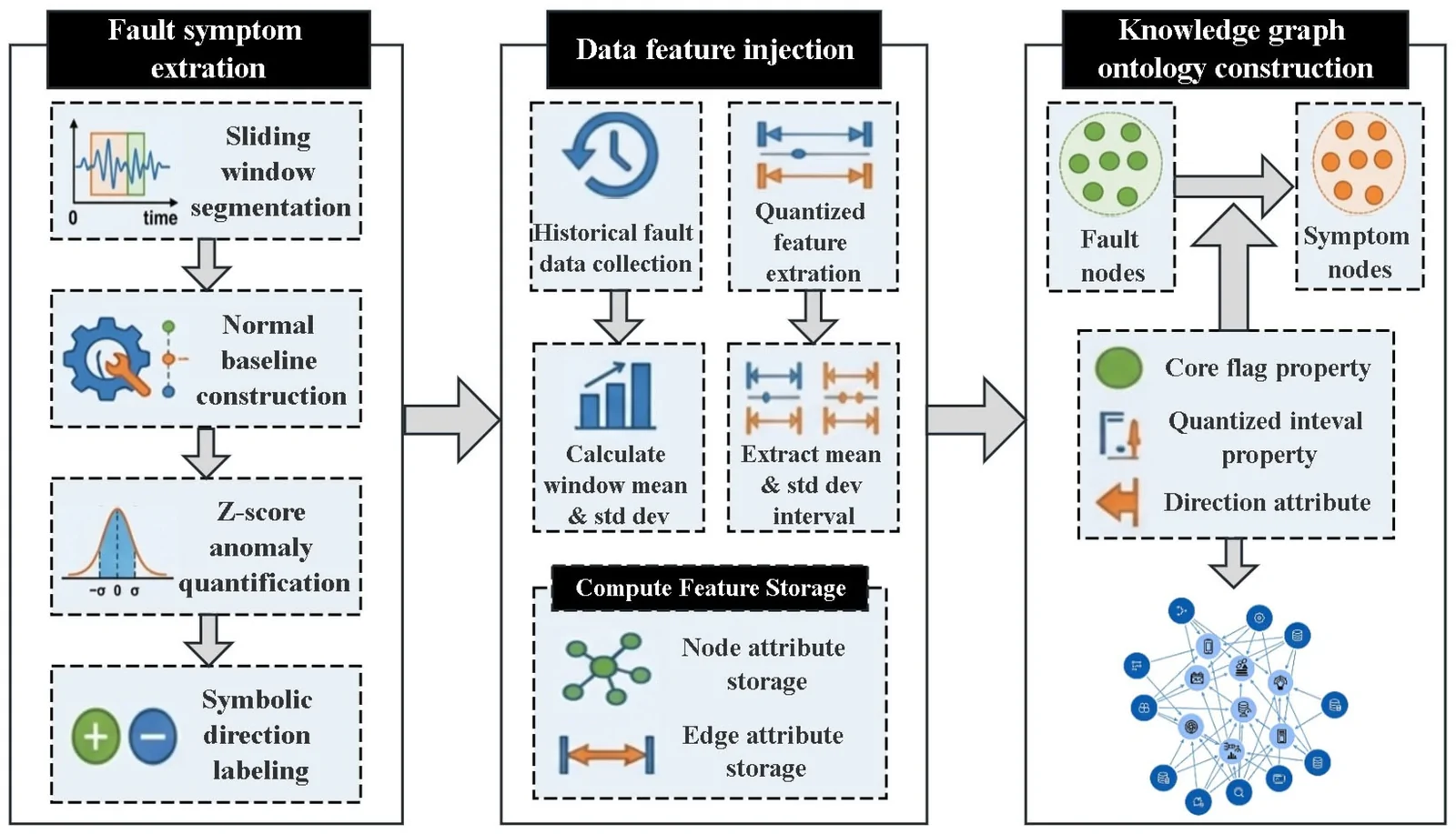
Flowchart of domain knowledge graph construction.

As a bridge connecting data and knowledge, a knowledge graph can explicitly transform fault experience, metric associations, and numerical constraints in distributed systems into computable structured knowledge [21]. Targeting the fault diagnosis scenario in distributed systems, this paper constructs a domain knowledge graph following the technical path of symptom extraction, feature injection and ontology modeling. In the first stage, through sliding window and Z-score anomaly detection, symbolic fault symptoms are extracted from raw monitoring time-series data, completing the conversion from numerical values to semantics [22]. In the second stage, based on symptom direction information, mean intervals and standard deviation intervals derived from historical fault data are injected into numerical nodes, endowing the graph with quantitative constraint capabilities [23]. Fault nodes, symptom nodes and relation edges are defined, clarifying causal associations and attribute specifications among entities. This knowledge graph organically integrates symbolic directional information with quantified numerical features, providing accurate and explicit knowledge support for subsequent LLM-based candidate filtering, causal propagation reasoning and confidence evaluation. The flowchart of this part is shown in Figure 2.

#### 2.1.1. Extraction of Fault Symptoms

Fault symptom extraction is a fundamental step in fault diagnosis for distributed systems. Its core objective is to identify and extract massive key indicator change patterns from raw time-series data which can characterize abnormal system states. In distributed systems, various performance metrics such as request processing rate, CPU usage, memory occupancy, and response latency are continuously generated. Under normal operating conditions, these metrics exhibit relatively stable distribution characteristics, whereas significant deviations occur when a fault happens [24]. Therefore, how to accurately capture these deviations from time-series data and transform them into structured fault symptoms directly affects the accuracy and interpretability of subsequent diagnosis.

Let the monitored system contain variables υ ∈ V and observations $x_{\nu }$(t). Because the injected faults manifest as sustained deviations rather than isolated spikes, each fault record is divided into non-overlapping windows of W = 10 consecutive observations. A normal baseline is estimated from fault-free observations for every variable. The standardized deviation of window k is defined by Equation (1), where the small constant ε prevents division by zero.

The symbolic anomaly direction is defined by Equation (2), with $\tau_{z}$ = 0.5 in the default configuration. Thus, the lower threshold is −0.5 rather than +0.5. The normal baseline is used only to standardize a diagnosed window; the class-specific mean and standard-deviation intervals stored in the KG are estimated exclusively from complete training windows.

(1) $$Z_{v}^{\left(k\right)}=\frac{{\bar{x}}_{v}^{\left(k\right)}-\mu_{v}^{N}}{\sigma_{v}^{N}+\varepsilon }$$

(2) $$d_{v}^{\left(k\right)}=\left\{\begin{array}{ll} +1, & Z_{v}^{\left(k\right)}>\tau_{z}\\ -1, & Z_{v}^{\left(k\right)}<-\tau_{z}\\ 0, & \left|Z_{v}^{\left(k\right)}\right|\leq \tau_{z} \end{array}\right.$$

This mapping converts continuous monitoring values into increased, decreased, or unchanged symptoms while retaining the original window statistics for subsequent interval checks. The resulting pair (${\bar{x}}_{v}^{\left(k\right)}$, $d_{v}^{\left(k\right)}$), together with the within-window standard deviation, forms the structured diagnostic input.

#### 2.1.2. Data Feature Injection

After the symbolic extraction of fault symptoms, this paper further injects quantitative data features into the knowledge graph to enhance the discriminative ability among different fault types. In real-world fault diagnosis scenarios, because multiple different fault types may produce the same direction of change on the same set of metrics and symptoms can be highly similar, relying solely on directional information for fault determination has obvious limitations [25].

To address this issue, this paper extracts the mean interval and standard deviation interval corresponding to each symptom under each fault type from historical fault data, injecting them into the knowledge graph. The mean interval reflects the actual numerical range of a symptom metric when a particular fault occurs [26]. For each fault and its corresponding symptom, by statistically processing the means of all windows in the training set of that fault, the minimum and maximum values are taken as the typical numerical interval of that symptom under that fault. This interval describes the general numerical level of the metric when the fault occurs. Even if different faults produce the same directional change on the same metric, their mean intervals may differ significantly, thereby providing a numerical basis for fault discrimination. The standard deviation interval further characterizes the degree of fluctuation of the metric during the fault process [27]. For each fault and its symptom, the standard deviation of that metric across all windows is calculated, and the minimum and maximum values are taken as the standard deviation interval of that symptom under that fault. For faults with highly consistent symptoms but different fluctuation characteristics, the standard deviation interval provides critical discriminative information.

The above two types of data features are computed and injected through automated scripts. Specifically, a window statistics script is used to partition the training data of each fault into windows, calculate the mean and standard deviation of each metric within each window, and then compute the mean interval and standard deviation interval for each symptom under each fault. These numerical values are then written into the numerical nodes of the knowledge graph, together with the symptom direction, training-derived interval attributes, and provenance information, forming a complete associative description from symptom to metric to fault. By injecting the mean intervals and standard deviation intervals, the knowledge graph is extended from a purely symbolic knowledge base to a numerical knowledge base incorporating quantitative constraints. In subsequent diagnostic reasoning, the system can use these numerical features to perform stricter filtering of candidate faults, laying the foundation for high-accuracy fault diagnosis.

#### 2.1.3. KG Ontology Construction

Based on fault symptom extraction and data feature injection, to integrate the scattered knowledge representations into a structured semantics, this paper constructs a domain knowledge graph for distributed system fault diagnosis [28]. This graph explicitly models the associations between fault types and symptom metrics, integrating the symbolic symptom directions and quantitative numerical features obtained in the previous steps, thereby providing explicit, traceable, and computable knowledge support for subsequent diagnostic reasoning [29].

The ontology consists of fault, symptom, and metric nodes linked by typed relations. Fault nodes represent the eight injected anomaly classes; symptom nodes represent observable directional changes; and metric nodes store the symptom name, description, direction, and class-specific mean and standard-deviation intervals. A symptom node is connected to a metric node via a HAS_METRIC relationship, and a metric node points to a fault node via an INDICATES_FAULT relationship [30]. Each edge from a metric node to a fault node is labeled by the corresponding fault class; this typed ontology follows established KG modeling and refinement principles rather than an image-recognition architecture.

In summary, the knowledge graph ontology constructed in this paper defines the semantic specifications of fault types, symptom metrics, and their associations. By incorporating attributes such as symptom descriptions, change directions, and mean and standard deviation intervals, it achieves a relatively comprehensive characterization of fault-symptom associations.

### 2.2. LLM-Based Iterative Diagnosis System

**Figure 3:**
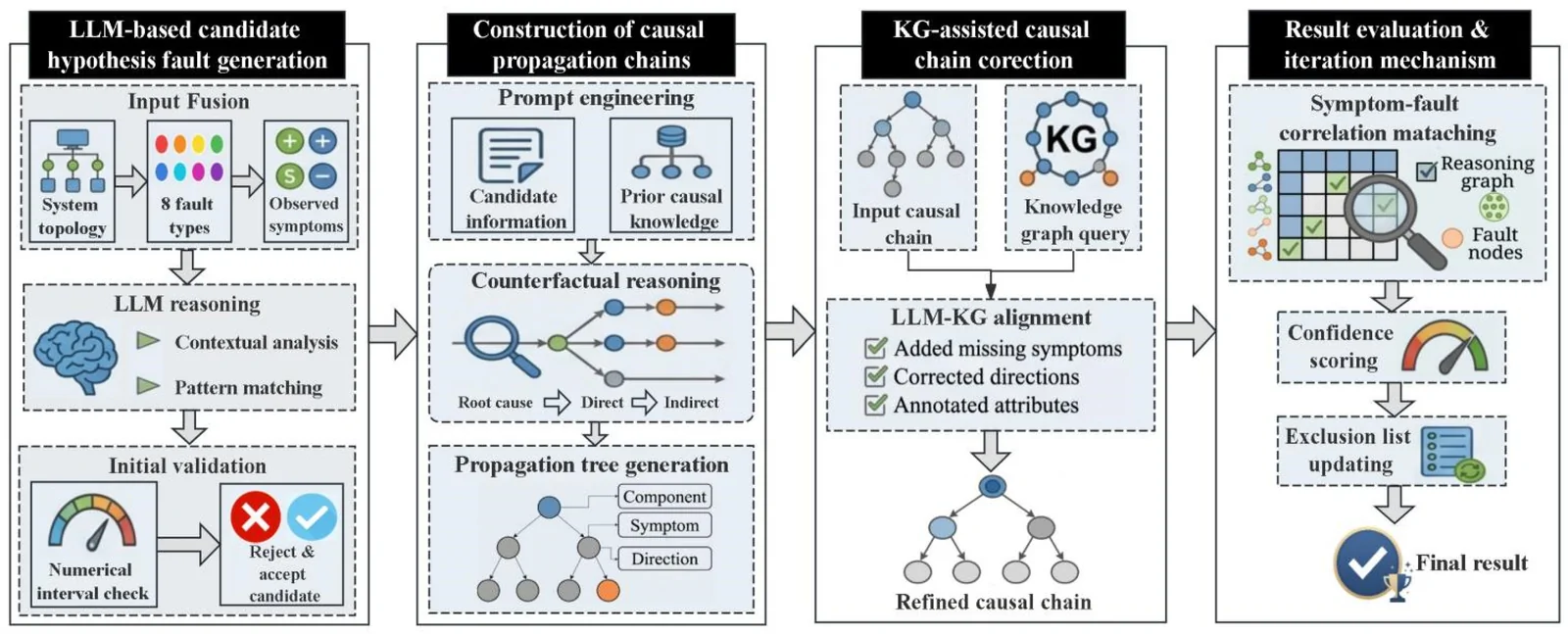
Flowchart of the LLM-based iterative diagnostic system.

This paper proposes an LLM-based iterative diagnosis system that simulates the cognitive loop of human experts: first perceiving system anomalies, then comprehending the observed symptoms, next reasoning along causal paths to form hypotheses, and finally verifying the hypotheses through evidence and iteration. The system diagram of this part is shown in Figure 3. The system takes the structured domain knowledge of the knowledge graph as its semantic foundation and the text understanding and multi-step reasoning capabilities of the LLMs as its core driving force, progressively approaching the root cause of the fault through an iterative elimination mechanism [31]. The LLM and the KG play complementary roles. The former provides flexible reasoning breadth, while the latter supplies precise domain constraints [32]. The two complement and validate each other, endowing the diagnostic results with high accuracy together with an explicit and interpretable reasoning trace.

#### 2.2.1. Candidate Fault Generation

In the LLM-based iterative diagnosis system proposed in this paper, prompt design is a key link connecting the LLM with system knowledge. This paper develops three prompt functions, which can automatically convert operational data into LLM input prompts in batches. Each prompt function contains several predefined slots, and the slot contents are dynamically filled by code at runtime, thereby ensuring consistency and reproducibility in interactions with the LLM. The specific slot definitions are shown in Table 1.

**Figure 4:**
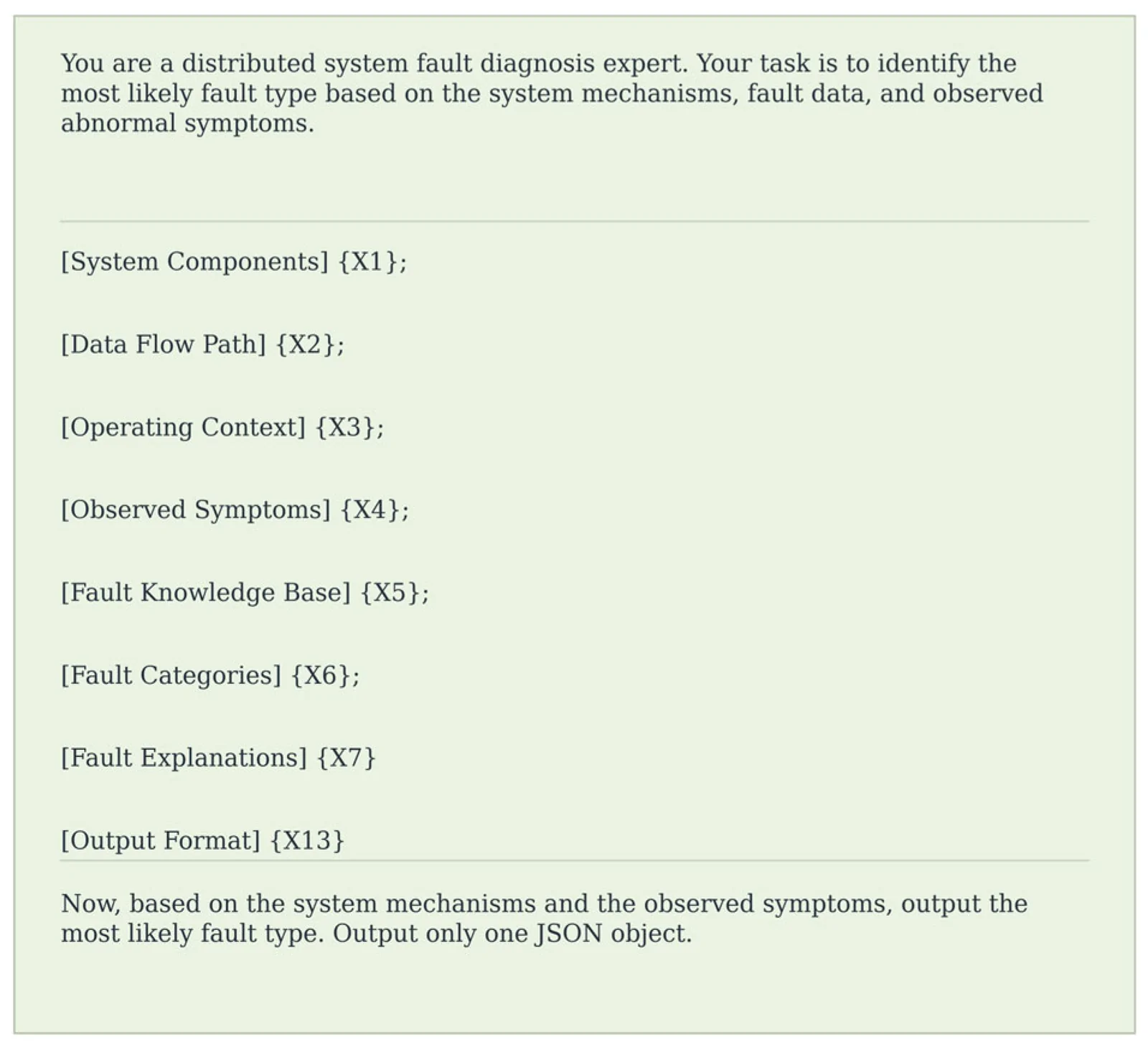
Illustration of the prompt function PF_candidate-generation_.

The prompt function in the first stage (PF_candidate-generation_) is used to guide the LLMs to generate candidate faults based on system mechanisms and fault knowledge, which is shown in Figure 4.

After candidate generation, the system retrieves the class-specific mean interval from the KG and expands it using the relative tolerance ρ, as defined in Equation (3), where ρ = 0.09 by default. A candidate that violates any required core interval is rejected, appended to the exclusion list, and not regenerated in the same diagnosis. This numerical gate precedes LLM causal reasoning and therefore limits unconstrained hypotheses.

(3) $$\begin{array}{rr} I_{v,c}\left(\rho \right) & =\left[m_{v,c}^{min}-\rho \Delta_{v,c},\;m_{v,c}^{max}+\rho \Delta_{v,c}\right]\\ \Delta_{v,c} & =m_{v,c}^{max}-m_{v,c}^{min} \end{array}$$

#### 2.2.2. Construction of Causal Propagation Chain

**Figure 5:**
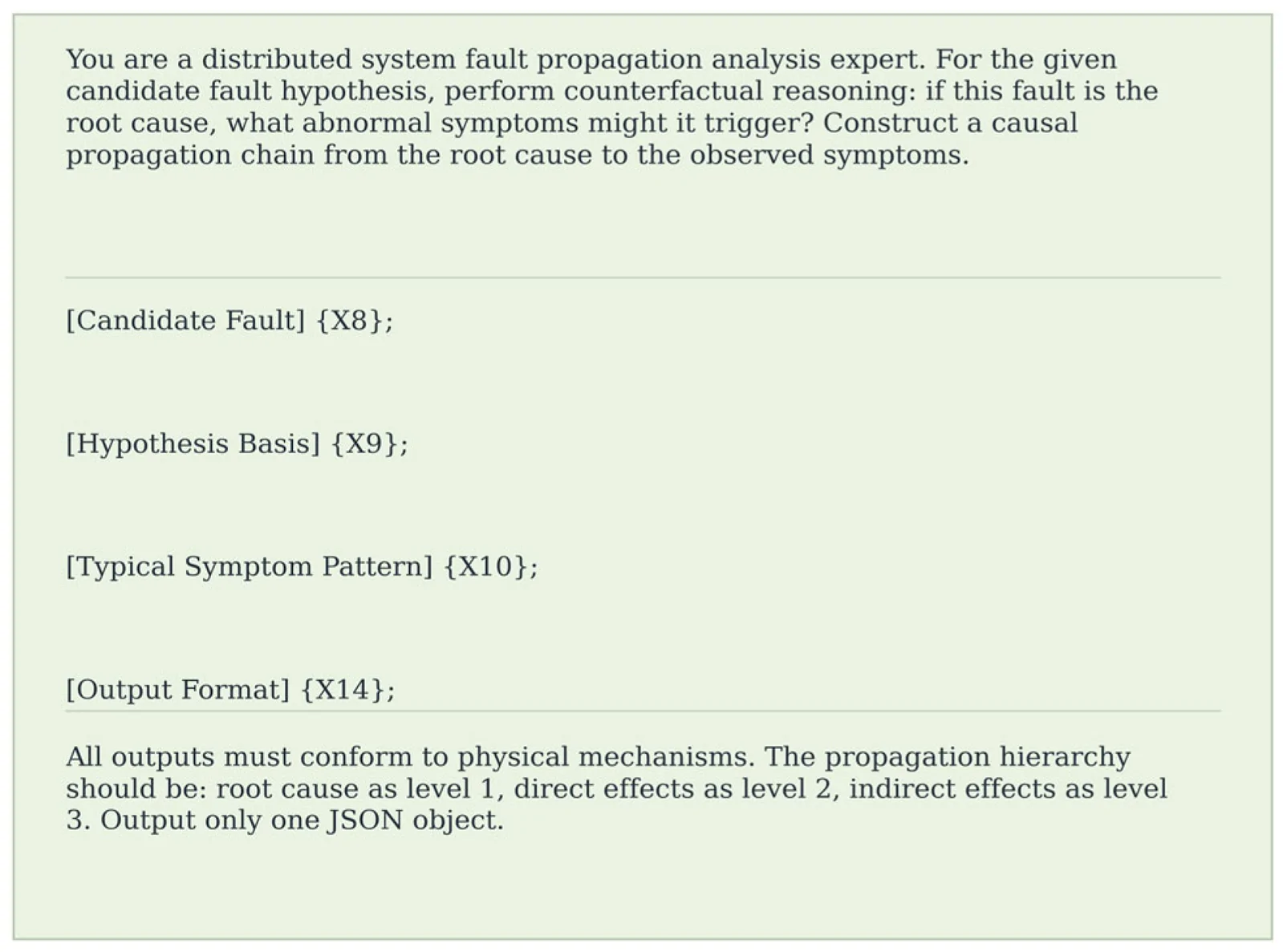
Illustration of the prompt function PF_counterfactual-reasoning_.

To simulate the process by which a human expert performs backward reasoning along the system causal chain after forming an initial fault hypothesis, this stage requires the LLMs to assume the fault as the root cause, counterfactually deduce its impact paths on various system components, and construct a hierarchical causal propagation chain from the root cause to the observed symptoms [33]. To achieve this goal, this paper designs the counterfactual reasoning prompt for the second stage (PF_counterfactual-reasoning_), as shown in Figure 5. The prompt first provides basic information about the candidate fault, including the fault type, name, and the reasoning basis from the first stage as the starting point for inference. Subsequently, the prompt incorporates the typical symptom pattern of the fault based on historical statistics, including symptoms and their expected directions of change. This part provides the LLMs with prior knowledge of fault propagation, guiding it to reason along plausible causal paths [34].

The prompt explicitly instructs the model to perform counterfactual reasoning. Assuming the candidate fault indeed occurs, the model infers the chain reaction it may trigger and constructs a propagation hierarchy from the root cause to direct effects and then to indirect effects [35]. To standardize the output format, the prompt requires a JSON structure containing the root cause description, the propagation tree and the effects at each level. Meanwhile, the prompt emphasizes that each variable appears only once in the propagation chain with a unique direction. If the same variable could have two possible changes, the most common direction is selected based on the typical statistical pattern of the fault, avoiding output ambiguity. In implementation, the LLMs perform layer-by-layer reasoning from the root cause based on the system’s physical mechanisms. Taking the candidate fault as the first layer, the model first infers which metrics of which components are directly affected, forming the second layer effects. On this basis, it further infers how these changes propagate to other components through inter-component dependencies, generating third and deeper indirect effects [36]. This hierarchical propagation structure is consistent with the physical connectivity of system components and facilitates subsequent verification and refinement.

This stage combines the causal reasoning capability of the LLMs with system physical knowledge, outputting a structured propagation chain that provides a clear prediction baseline for subsequent knowledge graph refinement and confidence verification. Through such counterfactual reasoning, the system can explore the propagation paths of faults in the hypothesis space, offering a causal basis for the reliability of the final diagnostic result.

#### 2.2.3. Refine the Causal Propagation Chain

Although an LLM possesses flexible causal-inference capabilities in counterfactual reasoning, it may miss certain variables or misjudge propagation paths due to insufficient understanding of specific system details. In contrast, the knowledge graph stores verified and precise associations between faults and symptoms, which can effectively correct and enhance the model output [37]. Therefore, using the structured domain knowledge pre-stored in the knowledge graph to supplement and refine the counterfactual propagation chain generated in the second stage can effectively improve the accuracy and completeness of the causal chain, thereby enhancing the diagnostic accuracy.

**Figure 6:**
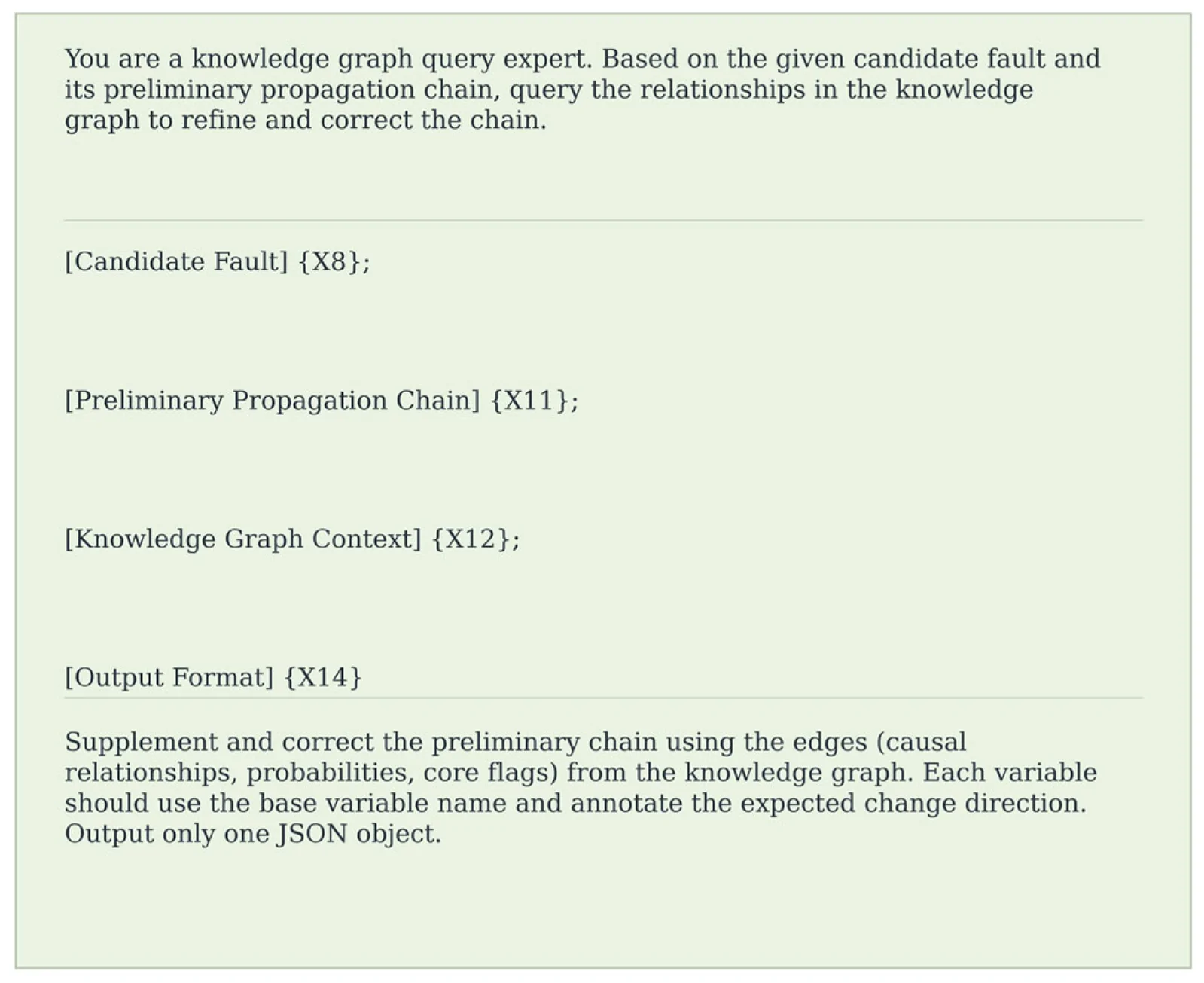
Illustration of the prompt function PF_refine-causal-chain_.

To this end, this paper designs the knowledge graph query prompt for the third stage (PF_refine-causal-chain_), as shown in Figure 6. The prompt first provides basic information about the candidate fault, including the fault type and name, serving as an identifier for the graph query. Subsequently, the prompt incorporates the preliminary propagation chain generated in the previous stage, presented in a structured format containing the root cause description, the propagation tree and the list of affected variables, providing a reference baseline for the model to refine the chain.

The prompt instructs the model to systematically supplement and refine the preliminary chain using the information retrieved from the knowledge graph. The specific tasks cover two aspects. The first is to add abnormal variables that exist in the graph but are missing from the preliminary chain, ensuring the completeness of the propagation chain. The second is to adjust the directions of variables that are inconsistent with the graph information, correcting possible reasoning biases. At the same time, the prompt emphasizes that the output format remains consistent with that of the second stage, containing the root cause description, the propagation tree and a summary statistic, to ensure input compatibility for the fourth stage verification. In implementation, the LLM first parses the list of variables in the preliminary propagation chain and then compares them item by item with the edge information in the graph. For symptoms that exist in the graph but are missing from the chain, the model supplements them into the corresponding level of the propagation tree. For variables whose directions in the chain are inconsistent with the graph, the model corrects them according to the graph information. The final output propagation chain not only retains the integrity of the model’s counterfactual reasoning but also incorporates the precise constraints of the knowledge graph, thereby improving the accuracy and robustness of the entire diagnosis system.

#### 2.2.4. Result Evaluation and Iteration

To simulate the verification and refinement process performed by human experts before reaching a final conclusion, this paper designs a fourth stage for result evaluation and iteration. The prompt first provides basic information about the candidate fault and then embeds the complete propagation chain refined in the third stage, including the predicted abnormal variables and their directions of change. At the same time, the prompt also injects the actually observed abnormal symptoms which are also presented in the form of symbolic directions. By presenting the predictions and the actual observations side by side, the prompt provides a clear basis for comparison.

The refined chain is compared with the observed symptoms variable by variable. Consistent directions are matches, opposite directions are conflicts, expected but unchanged variables are missing, and unpredicted observed anomalies are unexpected. The implementation uses the diagnostic matching score in Equation (4), rather than a calibrated probability. A candidate is accepted when the score is at least 0.60, and the search stops early when it reaches 1.85. The standard-deviation penalty and Fault 7 proximity term disambiguate the empirically similar Fault 5/Fault 7 patterns. The diagnostic matching score is a deterministic evidence score used for candidate acceptance and ranking; it is not interpreted as a calibrated posterior probability.

(4) $$\begin{array}{cc} S\left(c\right) & =0.45+0.20N_{match}-0.025N_{missing}-0.40N_{conflict}\\ & \;+0.20I\left(q_{initial}\geq 0.90\right)-0.30I_{std}+0.23I_{F7} \end{array}$$

The following numbered procedure summarizes the deterministic control logic. (1) Convert a complete 10-point case into window means, standard deviations, and symbolic directions. (2) Generate one non-excluded candidate and its initial confidence. (3) Reject the candidate if the KG mean-interval gate fails. (4) Construct a counterfactual causal chain and refine it with KG edges. (5) Compute $S\left(c\right)$, apply the standard-deviation rule, and accept candidates meeting the decision threshold. (6) Otherwise, add the candidate to the exclusion list and repeat, for at most five iterations. (7) If no candidate is accepted, return the candidate with the highest initial confidence. API responses are requested as JSON; Markdown fences are stripped, the first complete JSON object is extracted, trailing commas are repaired, and empty or malformed responses are retried at most twice and logged.

## 3. Results

### 3.1. Experimental System and Data Protocol

**Figure 7:**
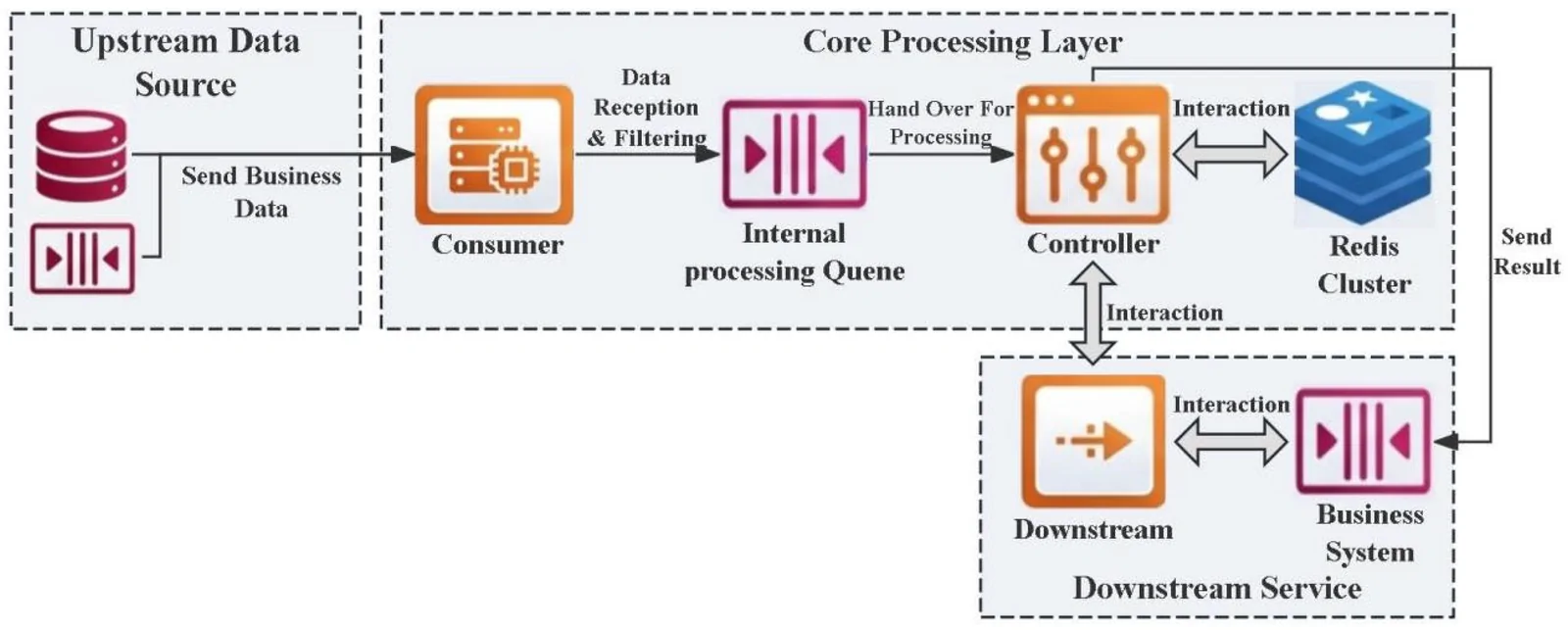
Architecture diagram of a distributed service system based on Redis.

This paper adopts a Redis-based distributed service system, as shown in Figure 7, as the experimental system. The architecture consists of a three-layer processing model comprising an upstream data source, a core processing layer and a downstream service. The upstream includes two external data channels, the sending end PW and the sending end PD, which are responsible for continuously sending business data to the system. The core processing layer includes the Consumer service, responsible for data reception and filtering, and the Controller service, which acts as the core scheduling component responsible for interacting with the Redis cluster and distributing data. The downstream service receives the data processed by the Controller and pushes it to the final business system.

The experimental dataset was constructed in a standardized distributed testing environment, using controlled variables and manual fault injection to simulate eight types of typical faults. The eight types of typical faults are shown in Table 2.

Monitoring data were sampled every 10 s for 18 variables covering service throughput, upstream/downstream latency, Redis and host resources, external data sources, errors, circuit-breaker state, and active instances. The definitions of these 18 variables are listed in Table 3. The fixed 7:3 partition was established at the fault-record level before window construction. Training and test records were windowed separately; no window crossed a partition boundary and no raw observation was reused across partitions. Numerical KG intervals were computed only from training windows. The 1724 training observations yield 172 complete 10-point windows, while the 744 test observations yield 68 complete diagnosis cases; the 64 trailing observations that do not form complete windows are excluded. Therefore, 744 denotes raw test observations and 68 denotes the evaluation denominator used by all methods. The detailed composition of the dataset and the case counts per fault class are shown in Table 4.

The per-class test supports are 10, 10, 7, 10, 7, 10, 7, and 7 for Faults 1–8, respectively. All baselines and KG + LLM variants use exactly these 68 cases. Accuracy is the proportion of correct cases; Macro-F1 averages classwise F1 equally, and Balanced Accuracy averages classwise recall. Accuracy confidence intervals are two-sided 95% Wilson intervals.

GPT-3.5, GPT-4o, and GPT-5.2 were accessed through APIYi’s OpenAI-compatible Chat Completions API (https://api.apiyi.com/v1) on 19 August 2026. Table 5 reports the model names passed in the code, the versions returned by the API, the shared generation settings, output limits, and retry behavior.

### 3.2. Results of KG Construction

Following the method described in Section 2.1, this paper constructs a domain knowledge graph for distributed-system fault diagnosis. The graph contains eight fault nodes, 11 directional symptom nodes, 48 numerical nodes, and eight solution nodes connected by typed relationships.

**Figure 8:**
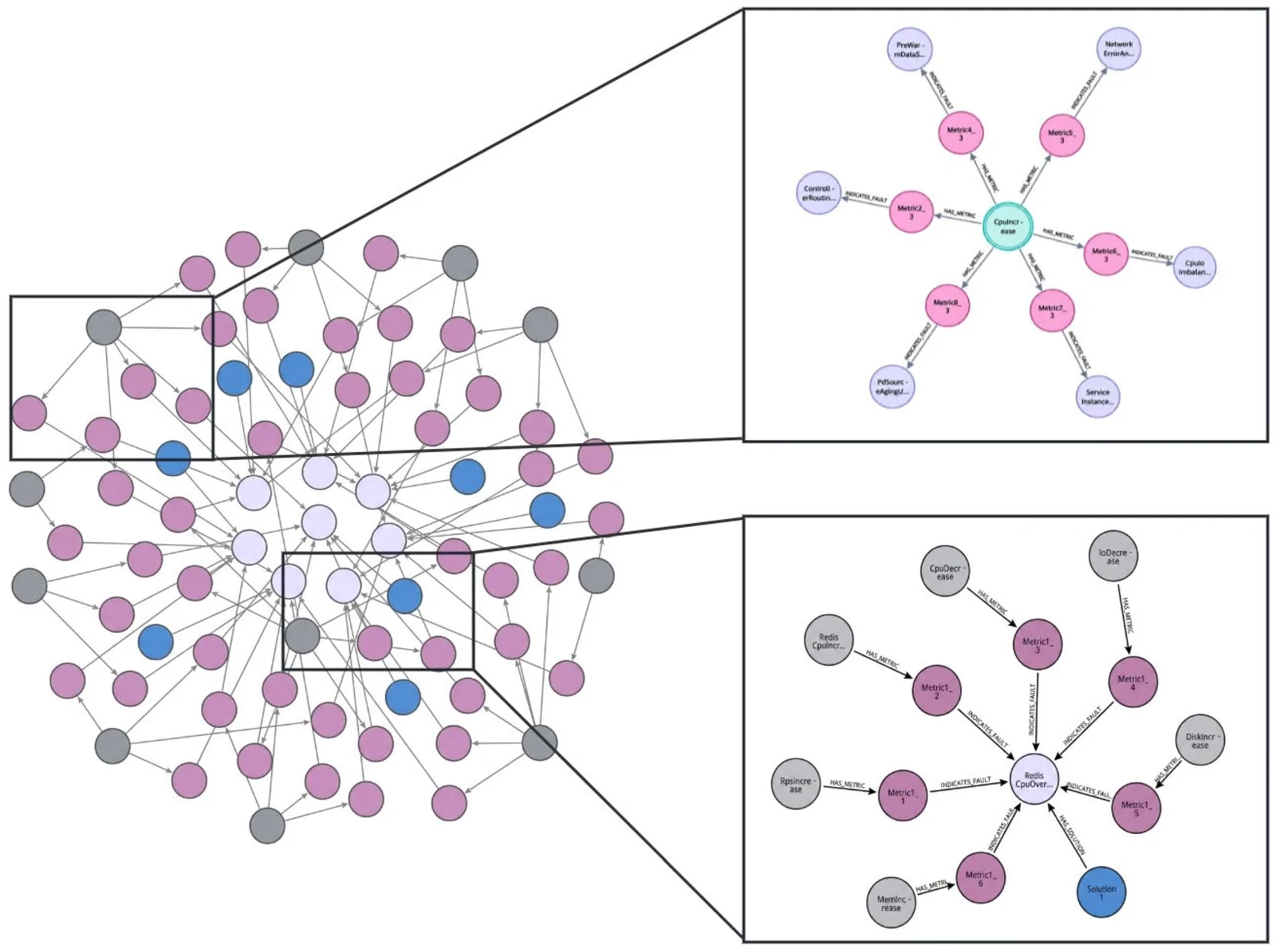
Diagram of knowledge graph for distributed system fault diagnosis.

The fault nodes cover the eight injected classes in Table 2. The 18 monitored variables in Table 3 are mapped to symptom and numerical nodes according to their diagnostic role; metrics that are informative in both directions are represented by separate increase/decrease symptom nodes, while numerical nodes store class-specific training-window mean and standard-deviation intervals. This explains why the graph contains 11 directional symptom nodes rather than one node for every raw variable. The KG is a strong, deterministic and traceable diagnostic foundation; the experiments below separately quantify its performance and the incremental contribution of LLM reasoning. The visual structure of the knowledge graph is shown in Figure 8.

### 3.3. Diagnostic Performance

#### 3.3.1. Diagnostic Results of the Proposed Fault Diagnosis Method

**Figure 9:**
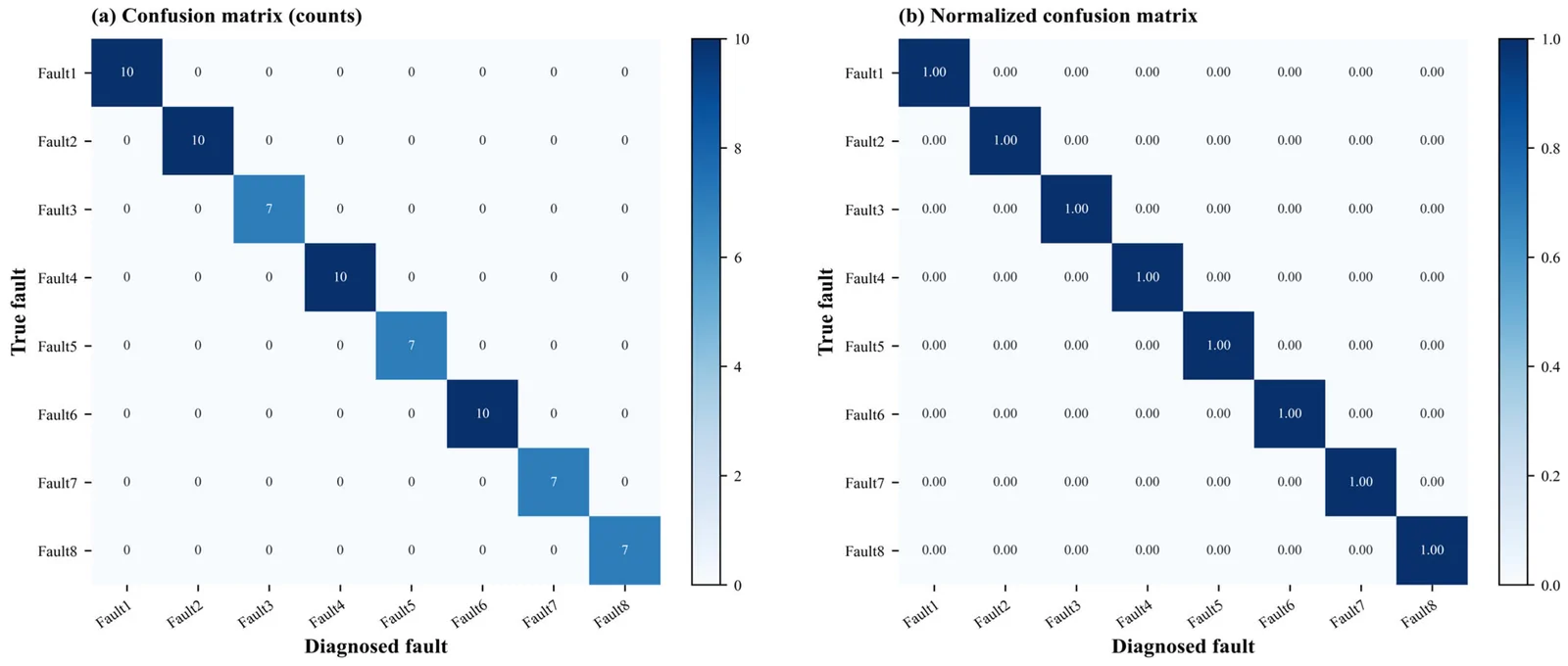
Count (**a**) and row-normalized (**b**) confusion matrices of the complete KG + GPT-4o configuration on 68 test cases; KG + GPT-5.2 produced the same result.

All methods were evaluated on the same 68 complete test cases. Figure 9 reports both the count and row-normalized confusion matrices for the complete GPT-4o configuration; GPT-5.2 produced the same matrix. The class supports are retained in the count panel, making the relationship between 744 raw observations and 68 diagnosis cases explicit.

All 68 cases are correctly classified, including the lower-support classes with seven cases. The perfect diagonal demonstrates separability within this controlled protocol; the Wilson interval in Table 6 is reported to avoid interpreting a finite-sample point estimate as population certainty.

#### 3.3.2. Comparative Experimental Results

Table 6 compares conventional learning, a temporal Transformer, KG-only reasoning, and complete KG + LLM configurations under the identical fixed split and 68-case denominator. Random Forest, XGBoost, and Transformer use the same window-level features and labels; KG-only applies the deterministic numerical and directional rules without an LLM.

Under the fixed 68-case protocol, the complete GPT-4o and GPT-5.2 configurations are the joint best methods, each achieving 68/68 correct cases and 100.00% on all three metrics. GPT-3.5 makes one error and reaches 98.53% accuracy. KG-only is a strong baseline at 91.18%, while Random Forest, Transformer, and XGBoost reach 89.71%, 88.24%, and 85.29%, respectively. The LLaMA configuration reaches 80.88%, indicating that an unreliable backbone can weaken rather than improve the deterministic KG decision. Table 7 reports the per-class precision, recall, and F1-score for the GPT-4o and GPT-5.2 configurations.

**Figure 10:**
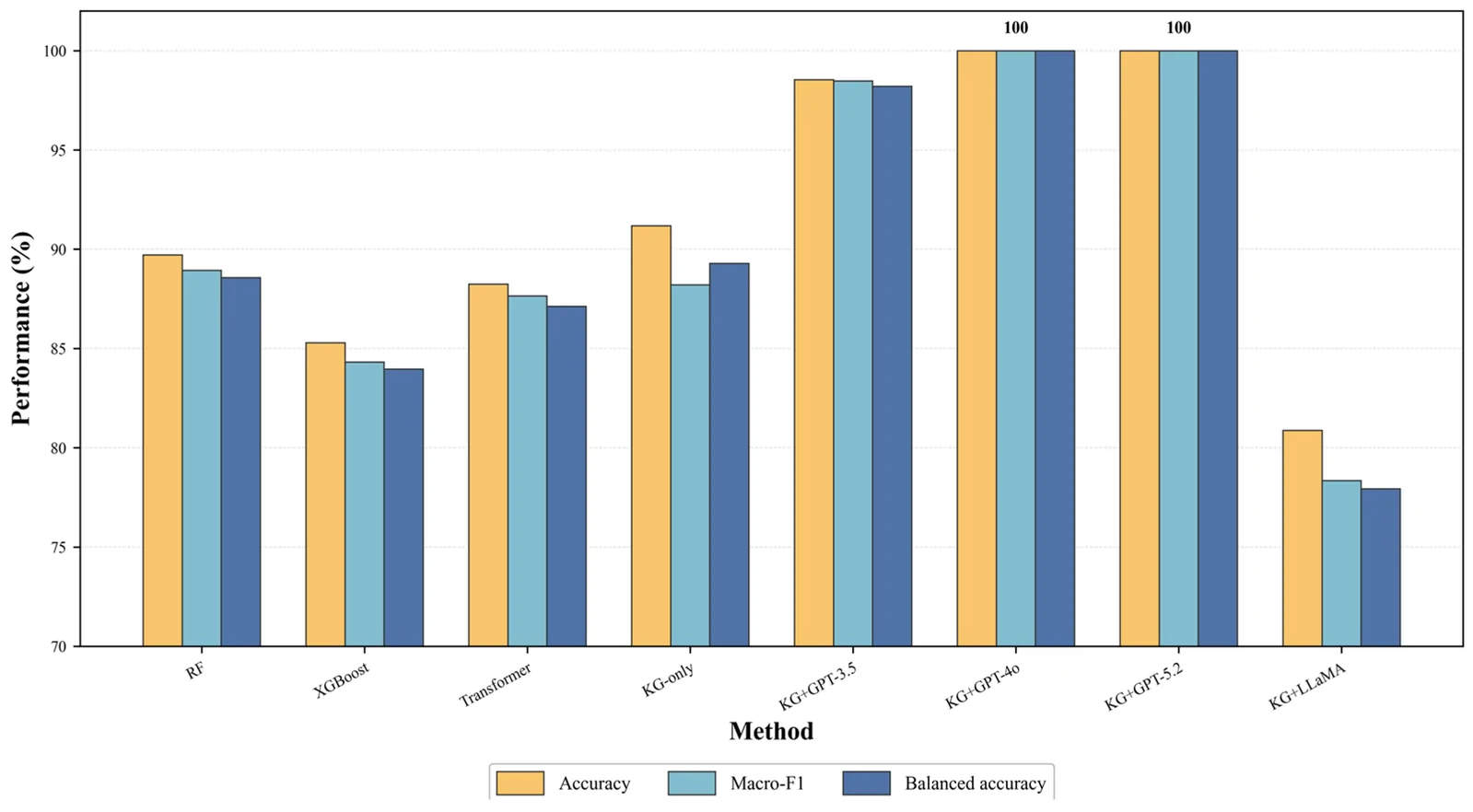
Overall Accuracy, Macro-F1, and Balanced Accuracy for the baseline and KG-enhanced configurations.

Figure 10 compares Accuracy, Macro-F1, and Balanced Accuracy across all eight methods. The two strongest configurations are tied at 100.00% on every metric, GPT-3.5 is close behind, and KG-only exceeds each conventional baseline in overall accuracy. The separation between the three metrics for LLaMA and KG-only also shows why accuracy alone is insufficient for an imbalanced per-class support pattern.

Repeated inference confirms that the two best configurations are stable under deterministic decoding: five GPT-4o runs and three GPT-5.2 runs each achieved 100.00% ± 0.00 for Accuracy, Macro-F1, and Balanced Accuracy, with zero errors per run. Parameter sensitivity was evaluated with GPT-4o at Z-score thresholds 0.3, 0.5, and 0.7; maximum iterations 1, 3, and 5; and relative tolerances 0.05, 0.09, and 0.15. Accuracy, Macro-F1, and Balanced Accuracy remained 100.00% at every evaluated setting. This stability supports the selected defaults within the tested ranges, without implying robustness to arbitrary parameter or distribution shifts.

#### 3.3.3. Ablation Experimental Results

The ablation study evaluates four configurations on the same 68 test cases: initial hypothesis only; iterative LLM reasoning without the KG; KG-only deterministic reasoning; and the complete KG + LLM framework. All four LLM backbones use the same data, prompt slots, and decision settings wherever the corresponding module is active.

Table 8 shows that the KG-only rules are already highly effective (91.18%). Adding reliable LLM reasoning yields further gains for GPT-3.5 (+7.35 percentage points) and GPT-4o/GPT-5.2 (+8.82 points). By contrast, complete KG + LLaMA reaches 80.88%, which is 10.30 points below KG-only but 23.53 points above iterative LLaMA reasoning without the KG. Thus, the KG contributes substantial constraint value for every backbone, whereas a positive gain over KG-only depends on backbone reliability.

**Figure 11:**
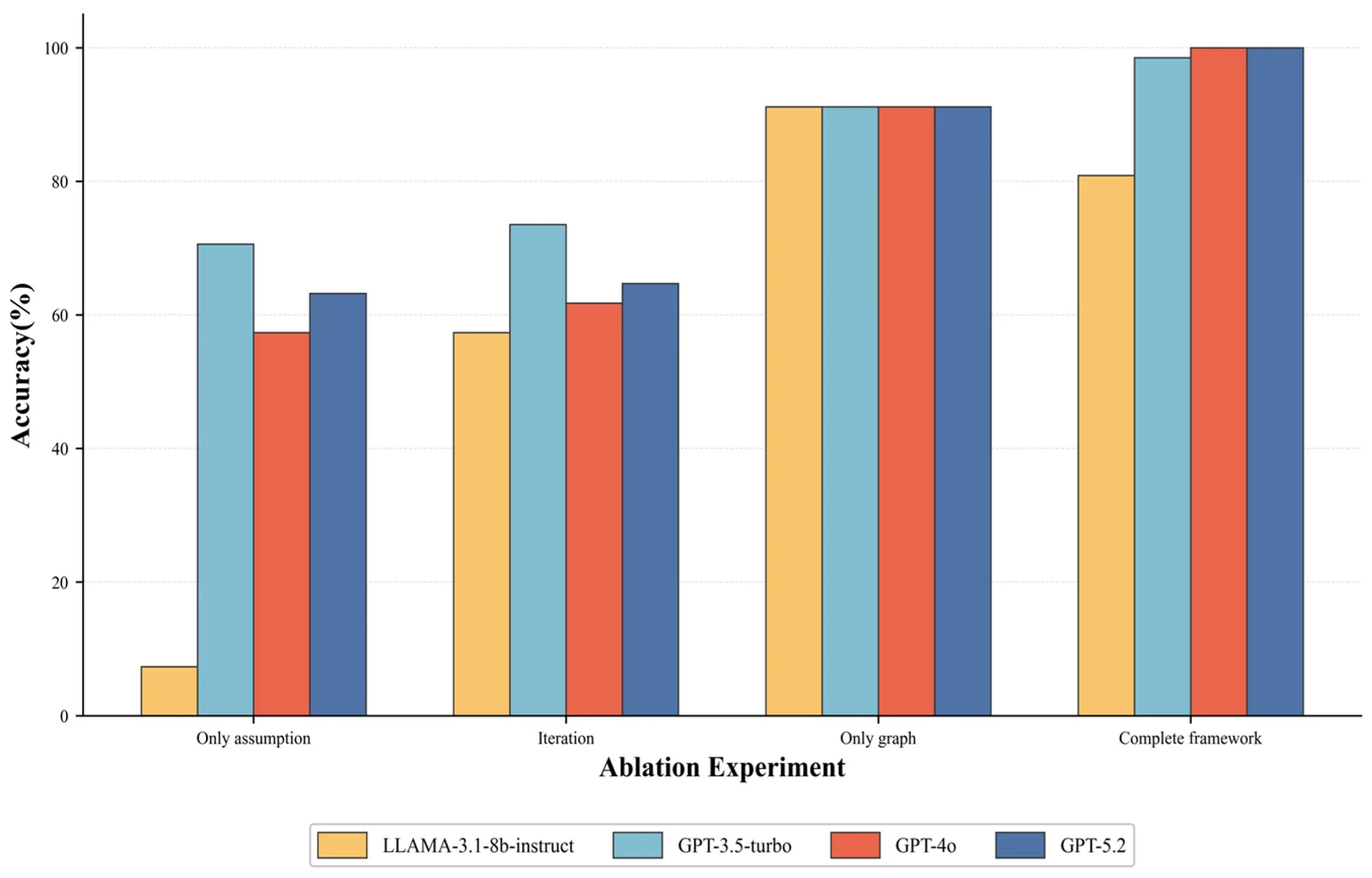
Accuracy of four ablation configurations across the evaluated LLM backbones.

Figure 11 visualizes the same ablation. Initial hypotheses and unconstrained iterative LLM reasoning are insufficient for this task. KG constraints produce the largest consistent improvement, while stronger GPT backbones add effective disambiguation and causal evidence integration. The LLaMA result is intentionally retained: it defines an important applicability condition and prevents the inaccurate claim that every LLM necessarily improves a strong KG-only baseline.

### 3.4. Prototype Interaction Demonstration

**Figure 12:**
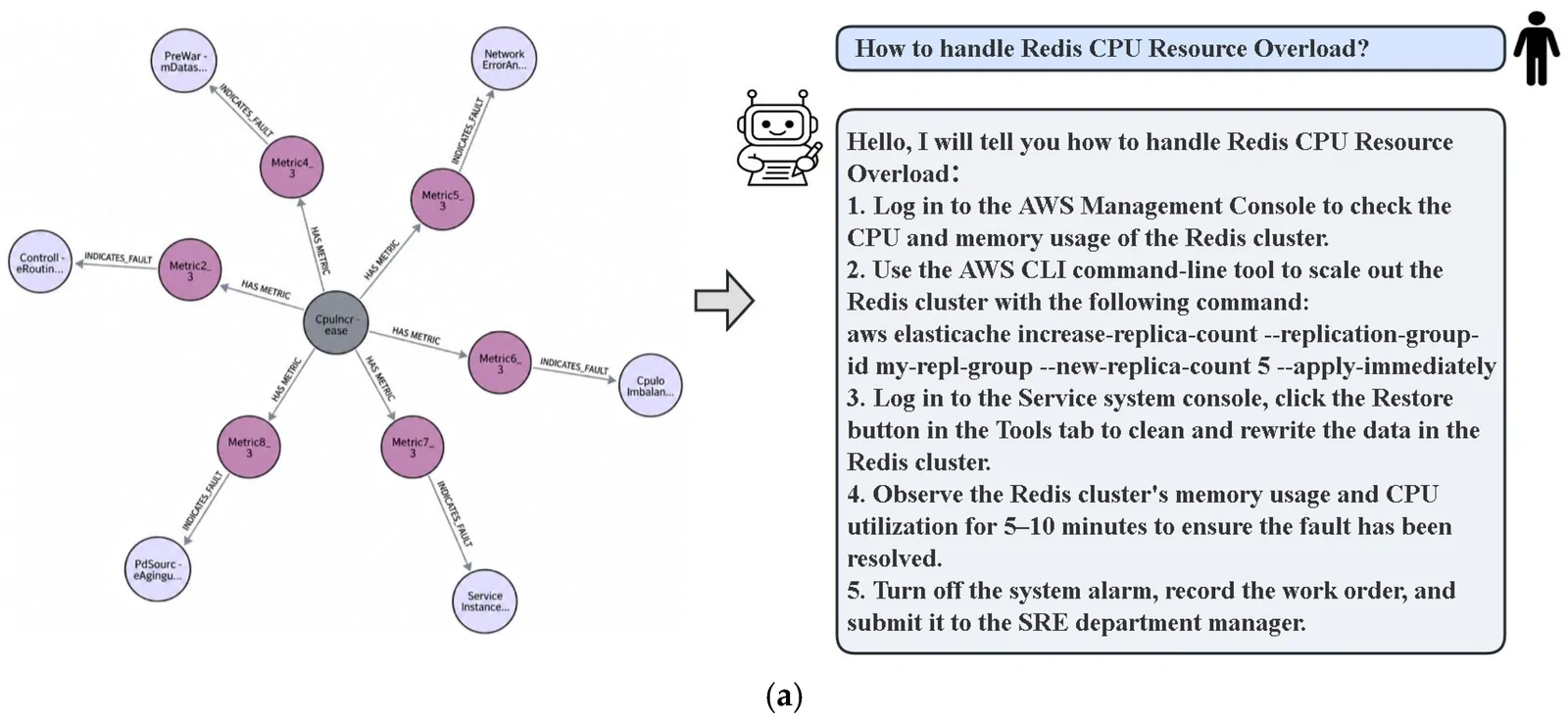

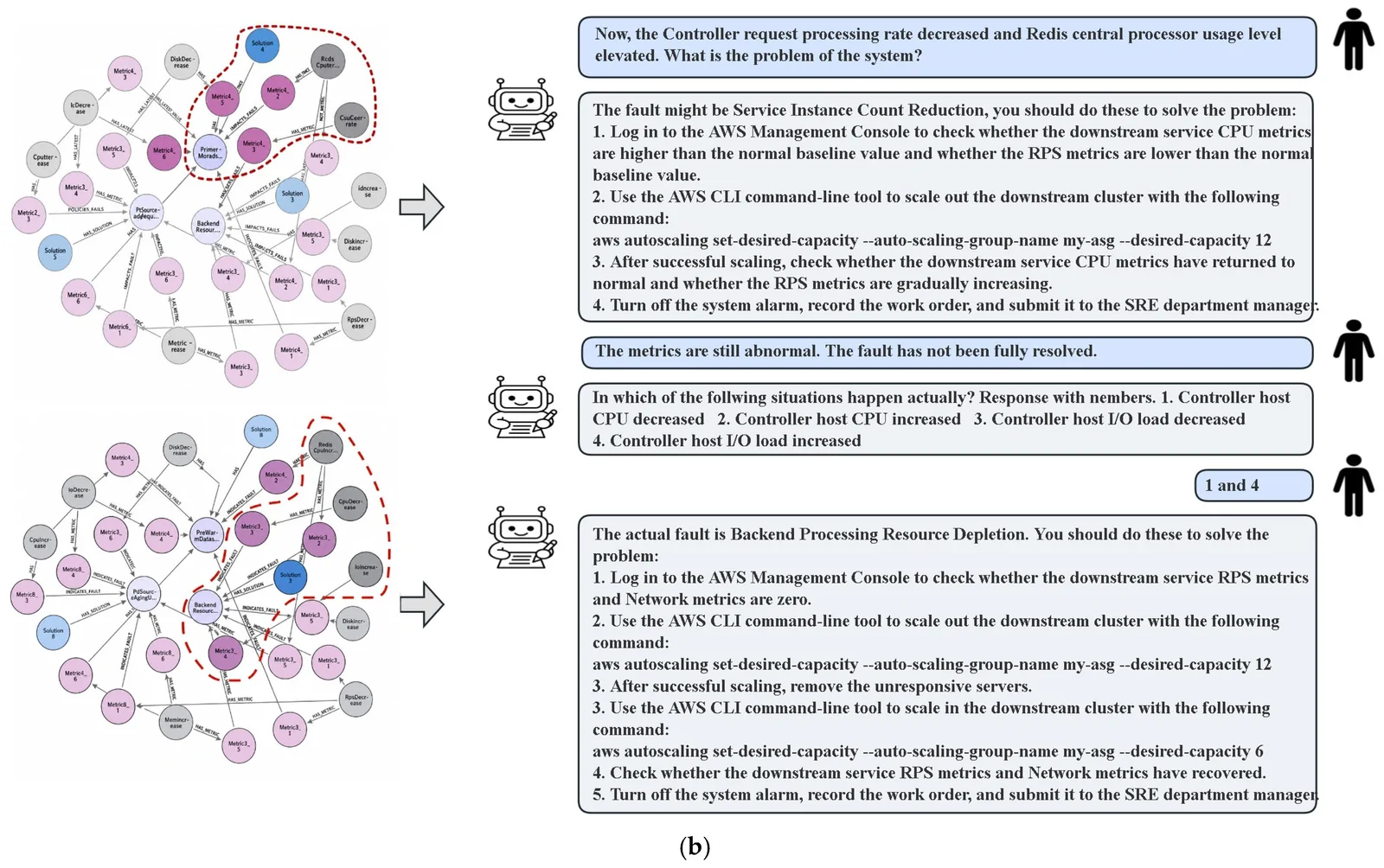
Prototype interaction based on the KG. (**a**) Single-round KG traversal and solution retrieval. (**b**) Multi-round feedback-based diagnostic workflow.

To demonstrate how the diagnostic outputs can support operator interaction, the prototype maps natural-language or structured symptom input to KG entities, traverses candidate fault and solution relations, and accepts subsequent operator feedback. Figure 12 illustrates the implemented interaction flow; it is a functional demonstration rather than a controlled user study.

As illustrated in Figure 12a, when a user inputs fault information through natural language or a structured form, the system first uses the LLMs for entity recognition and intent parsing, mapping the user input to an initial set of nodes in the graph. Subsequently, taking these nodes as starting points, the system initiates a directed graph traversal according to the predefined relationships in the graph. During the traversal, the system locates the most probable root cause nodes through the associative relationships in the graph and simultaneously retrieves the “solution” nodes connected to the root cause nodes, providing corresponding fault solutions in the actual interaction.

Figure 12b shows the feedback loop. After an initial candidate and solution are returned, an operator can report resolved, partially resolved, or ineffective outcomes and provide additional symptoms. The new evidence triggers another constrained traversal and diagnosis cycle. A formal human-in-the-loop evaluation with interaction logs, diagnosis time, and operator feedback is reserved for future deployment studies.

## 4. Discussion

### 4.1. Evidence-Supported Advantages

The experiments identify three complementary sources of performance. First, KG-only reasoning reaches 91.18%, showing that train-derived numerical intervals and directional relations form a strong deterministic basis. Second, reliable LLM backbones add candidate disambiguation, contextual evidence synthesis, and explicit causal-chain construction: GPT-3.5, GPT-4o, and GPT-5.2 improve on KG-only by 7.35, 8.82, and 8.82 percentage points, respectively. Third, every stage exposes traceable intermediate output—candidate hypotheses, interval decisions, propagation chains, KG corrections, and matching scores—rather than returning only a class label.

The repeated-run and sensitivity results provide additional evidence of stability within the evaluated protocol. GPT-4o and GPT-5.2 remain perfect across their repeated executions, and GPT-4o remains unchanged under moderate perturbations of the Z-score threshold, maximum iterations, and interval tolerance. This supports highly accurate, stable, and knowledge-constrained diagnosis within the fixed 68-case Redis protocol.

The ablation also establishes a clear boundary: LLaMA-3.1-8B improves over its unconstrained reasoning configuration but remains below KG-only. Positive KG–LLM synergy is therefore backbone-dependent, and the deterministic KG should remain available as a safety-oriented fallback when model reasoning is unreliable.

### 4.2. Limitations and Future Research Directions

The current evaluation focuses on one Redis-based architecture and eight known single-root-cause fault classes under controlled injection. The 100% point estimates describe performance on these 68 complete test cases. Cross-system, cross-workload, unseen-fault, and concurrent multi-fault generalization remain to be evaluated. Future work will add a second distributed-service platform, grouped fault-event evaluation under workload shift, explicit unknown-fault rejection, and multi-hypothesis reasoning for overlapping propagation chains.

The numerical KG is currently built offline. As services, metrics, and operating regimes evolve, its intervals and relations may require revision. A deployment-oriented extension should combine drift detection, expert-verified incremental graph updates, and versioned provenance so that every numerical constraint remains auditable.

Finally, traceability is demonstrated through explicit intermediate reasoning artifacts, but operator benefit has not been quantified. The interaction prototype should be evaluated with diagnosis time, correction frequency, solution usefulness, and structured operator feedback before making claims about maintenance efficiency or usability.

Request-level latency, token usage, and API cost were not recorded for the final evaluation, so reliable numerical estimates for these engineering quantities cannot be reconstructed retrospectively. Future deployment benchmarking should record these measures under fixed model and API configurations.

## 5. Conclusions

This paper presents a knowledge-enhanced iterative reasoning framework for fault diagnosis in distributed service systems. A numerical KG encodes fault–symptom relations, anomaly directions, and training-derived mean and standard-deviation intervals. Four stages—candidate generation and interval verification, counterfactual causal-chain construction, KG refinement, and deterministic evidence scoring with iterative exclusion—form a traceable route from monitoring observations to root-cause decisions.

Under the common 68-case protocol for eight known single-root-cause faults in the controlled Redis-based testbed, KG + GPT-4o and KG + GPT-5.2 achieve 100.00% Accuracy, Macro-F1, and Balanced Accuracy, compared with 91.18% accuracy for KG-only and 85.29–89.71% for the conventional and Transformer baselines. GPT-3.5 reaches 98.53%. Repeated runs of the two best configurations yield 100.00% ± 0.00, and all tested sensitivity settings remain at 100.00%. These results support high accuracy and stability under the tested conditions.

The ablation study further separates the contributions of numerical knowledge and LLM reasoning. Reliable GPT backbones improve on the strong KG-only baseline, while LLaMA-3.1-8B remains below KG-only, demonstrating that the incremental benefit is model-dependent. The framework retains a high standard of traceability because candidate, causal-chain, KG-correction, and score evidence can be inspected at every stage. The prototype interaction and solution-retrieval workflow show deployment-oriented potential; cross-system, unseen-fault, multi-fault, and human-in-the-loop evaluations remain the next steps toward broader operational validation.

## Figures and Tables

**Table 1:** Definition of prompt slot.

Slot	Stage	Meaning
X_1_	1	List of main system components
X_2_	1	Data flow path relationships
X_3_	1	Operational context
X_4_	1	Actual observed symptoms
X_5_	1	Fault knowledge base
X_6_	1	List of fault categories
X_7_	1	Fault explanation
X_8_	2, 3	Candidate fault information
X_9_	2	Hypothesis basis
X_10_	2	Typical symptom pattern
X_11_	3	Preliminary propagation chain
X_12_	3	Knowledge graph edge information
X_13_	1	Output format of the first stage
X_14_	2, 3	Output format of stages 2 and 3

**Table 2:** Eight fault types of distributed service systems based on redis.

Fault Types	Fault Name
Fault 1	Redis CPU resource overload
Fault 2	Controller routing configuration issue
Fault 3	Backend processing resource depletion
Fault 4	PreWarm data source interruption
Fault 5	Network error count anomaly
Fault 6	CPU-IO imbalance with circuit breaker activation
Fault 7	Service instance count reduction
Fault 8	PD source aging and upstream latency anomaly

**Table 3:** Definition of the 18 monitored variables.

Variable	Component/Domain	Definition
RPS_S	Controller/service	Request-processing rate at the service/controller side
UP_LT	Upstream path	Upstream communication latency
CPU_RE	Redis cluster	Redis CPU utilization
ERR_COUNT	Network/service	Number of observed request or network errors
preWarm	PreWarm source	PreWarm-source measurement/value
product	Product source	Product-source measurement/value
onHandPrice	On-hand-price source	On-hand-price source measurement/value
OH_NUM	On-hand-price source	Number of on-hand-price records/messages
PW_NUM	PreWarm source	Number of PreWarm records/messages
PD_NUM	PD source	Number of PD-source records/messages
CPU	Controller host	Host CPU utilization
IO	Controller host	Host input/output load
RPS_H	Downstream/host service	Request-processing rate at the host/downstream side
INS	Service deployment	Number of active service instances
DISK	Controller host	Host disk utilization/load
DS_CB	Downstream path	Downstream circuit-breaker state or activation indicator
DS_LT	Downstream path	Downstream communication latency
MEM	Controller host	Host memory utilization

**Table 4:** Dataset composition and evaluation units.

Fault	Train Obs.	Test Obs.	Train Windows	Test Cases	Unused Remainder
Fault 1	251	108	25	10	8
Fault 2	251	108	25	10	8
Fault 3	180	78	18	7	8
Fault 4	251	108	25	10	8
Fault 5	180	78	18	7	8
Fault 6	251	108	25	10	8
Fault 7	180	78	18	7	8
Fault 8	180	78	18	7	8
Total	1724	744	172	68	64

**Table 5:** Algorithmic and LLM reproducibility settings.

Category	Setting	Value	Role/Implementation
Data	Window/sampling	10 observations/10 s	One diagnosis case per complete window
Decision	Z threshold/tolerance	±0.5/0.09	Symbolization and KG interval expansion
Decision	Max. iterations	5	Candidate-generation and verification cycles
Score	Base; match; missing; conflict	0.45; +0.20; −0.025; −0.40	Diagnostic matching score terms
Score	High initial confidence	+0.20 at q ≥ 0.90	Initial-hypothesis bonus
Score	Std violation/Fault 7 rule	−0.30/+0.23	Fault 5/Fault 7 disambiguation
Decision	Accept/early stop	0.60/1.85	Acceptance and termination thresholds
Decision	Fallback	Highest initial confidence	Used if no candidate passes
LLM	GPT-3.5 (request/returned)	gpt-3.5-turbo/gpt-3.5-turbo-0125	APIYi; 19 August 2026
LLM	GPT-4o (request/returned)	gpt-4o/gpt-4o-2024-11-20	APIYi; 19 August 2026; 5 repetitions
LLM	GPT-5.2 (request/returned)	gpt-5.2/gpt-5.2-2025-12-11	APIYi; 19 August 2026; 3 repetitions
LLM	Shared GPT API settings	Temperature: 0.0; top-p: not explicitly set; seed: not explicitly set	For all three GPT models; top-p uses interface backend default; seed exact value not recorded or returned
LLM	LLaMA-3.1-8B	llama-3.1-8b-instruct	Temperature 0; provider-default top-p
Output	Token limits	JSON: 3000; ordinary text: 1500	Maximum output tokens per request
Output	Malformed response	At most two retries	Fence removal, JSON extraction, comma repair, logging

**Table 6:** Baseline and overall diagnostic performance under the common 68-case test protocol.

Method	Correct	Accuracy (%)	Macro-F1 (%)	Balanced Accuracy (%)	Accuracy 95% CI (%)
Random Forest	61/68	89.71	88.94	88.57	80.24–94.92
XGBoost	58/68	85.29	84.31	83.96	75.00–91.81
Transformer	60/68	88.24	87.65	87.12	78.47–93.92
KG-only	62/68	91.18	88.21	89.29	82.06–95.89
KG + GPT-3.5	67/68	98.53	98.47	98.21	92.13–99.74
**KG + GPT-4o**	**68/68**	**100.00**	**100.00**	**100.00**	**94.65–100.00**
**KG + GPT-5.2**	**68/68**	**100.00**	**100.00**	**100.00**	**94.65–100.00**
KG + LLaMA-3.1-8B	55/68	80.88	78.35	77.94	69.99–88.70

**Table 7:** Per-class results for GPT-4o and GPT-5.2 under the 68-case test protocol.

Fault	Support	GPT-4o P	GPT-4o R	GPT-4o F1	GPT-5.2 P	GPT-5.2 R	GPT-5.2 F1
Fault 1	10	1.000	1.000	1.000	1.000	1.000	1.000
Fault 2	10	1.000	1.000	1.000	1.000	1.000	1.000
Fault 3	7	1.000	1.000	1.000	1.000	1.000	1.000
Fault 4	10	1.000	1.000	1.000	1.000	1.000	1.000
Fault 5	7	1.000	1.000	1.000	1.000	1.000	1.000
Fault 6	10	1.000	1.000	1.000	1.000	1.000	1.000
Fault 7	7	1.000	1.000	1.000	1.000	1.000	1.000
Fault 8	7	1.000	1.000	1.000	1.000	1.000	1.000
Macro average	68	1.000	1.000	1.000	1.000	1.000	1.000

**Table 8:** Ablation-study accuracy (%).

Configuration	LLaMA-3.1-8B	GPT-3.5	GPT-4o	GPT-5.2
Initial hypothesis only	7.35	70.59	57.35	63.24
Iterative LLM reasoning without KG	57.35	73.52	61.76	64.71
KG-only	**91.18**	91.18	91.18	91.18
Complete KG + LLM framework	80.88	**98.53**	**100.00**	**100.00**

## Data Availability

The dataset and source code supporting the findings of this study are publicly available on Figshare under the title “Distributed systems” at https://doi.org/10.6084/m9.figshare.32991179. The archive includes the monitoring dataset, fixed training–test partitions, preprocessing scripts, knowledge-graph construction files, prompt templates, model configurations, and evaluation scripts.
